# Systematic Review on Information Technology Approaches to Evaluate the Impact of Public Health Campaigns: Real Cases and Possible Directions

**DOI:** 10.3389/fpubh.2021.715403

**Published:** 2022-01-11

**Authors:** Rafael Pinto, Lyrene Silva, Ricardo Valentim, Vivekanandan Kumar, Cristine Gusmão, Carlos Alberto Oliveira, Juciano Lacerda

**Affiliations:** ^1^Department of Informatics and Applied Mathematics, Federal University of Rio Grande do Norte, Natal, Brazil; ^2^Laboratory of Technological Innovation in Health (LAIS), Federal University of Rio Grande do Norte, Natal, Brazil; ^3^Information Systems Coordination, Federal Institute of Rio Grande do Norte, Natal, Brazil; ^4^Department of Biomedical Engineering, Federal University of Rio Grande do Norte, Natal, Brazil; ^5^School of Computing and Information Systems, Athabasca University, Athabasca, Canada; ^6^Department of Biomedical Engineering, Federal University of Pernambuco, Recife, Brazil; ^7^Multidisciplinary Department of Human Development with Technologies, State University of Rio de Janeiro, Rio de Janeiro, Brazil; ^8^Department of Social Communication, Federal University of Rio Grande do Norte, Natal, Brazil

**Keywords:** public health, campaign, evaluation, systematic review, communicable disease

## Abstract

Evaluating the success of a public health campaign is critical. It helps policy makers to improve prevention strategies and close existing gaps. For instance, Brazil's “Syphilis No!” campaign reached many people, but how do we analyze its real impact on population awareness? Are epidemiologic variables sufficient? This study examined literature on using of information technology approaches to analyze the impact of public health campaigns. We began the systematic review with 276 papers and narrowed it down to 17, which analyzed campaigns. In addition to epidemiological variables, other types of variables of interest included: level of (i) access to the campaign website, (ii) subject knowledge and awareness, based on questionnaires, (iii) target population's interest, measured from both online search engine and engagement with Social Network Service, and (iv) campaign exposure through advertising, using data from television commercials. Furthermore, we evaluated the impact by considering several dimensions such as: communication, epidemiology, and policy enforcement. Our findings provide researchers with an overview of various dimensions, and variables-of-interest, for measuring public campaign impact, and examples of how and which campaigns have used them.

## Introduction

Public communications campaigns (PCC) are strategic to communicating important information to the public (1). In the context of this work, PCCs for Health Care will be called public health campaigns (PHC). PHC aims to influence behavior either on an individual level, through direct messages, or collectively with policies that inspire change (1).

PHC aims to promote awareness, increase knowledge, encourage the adoption of desirable attitudes and behaviors, and contribute to individual and collective health decision-making (2). PHCs are often sponsored by policy makers, and offer preventive recommendations addressing serious health problems, such as sexually transmitted diseases, alcohol, tobacco, and obesity, as well as the dangers of automobiles, guns and pharmaceuticals (3).

For example, the “Sífilis Não!” (Syphilis No!) campaign, communicated the importance of syphilis prevention, and recommended rapid syphilis testing or VDRL test, conducted at Basic Health Units (UBS) of the Unified Health System (SUS) in Brazil. The Ministry of Health sponsored the campaign, to fight the syphilis epidemic in the country. Between Nov 2018 and May 2019, a variety of content reached large audiences through various media platforms (e.g., television, radio, and online), as well as outdoor media, such as billboards and posters, and print media, such as magazines and newspapers.

Campaign evaluations serve several useful purposes. They help policy makers improve prevention strategies and close existing gaps. Analysis can guide the development of better campaigns, to improve the impact or intensify the message. Understanding the effectiveness of a campaign can help to better direct the use of funds and effort. To assess levels of engagement with a given topic on a platforms such as the Internet, researchers are increasingly using computational approaches. A variety of such techniques and technologies have been used to assess the impact of public health campaigns. Subsequently, a secondary study is beneficial both to understand the current state-of-the-art and to guide future research in this domain.

Accordingly, we performed a systematic literature review (SLR) according to Kitchenham and Charters (4) and Petersen et al. (5) to: (1) characterize scientific literature related to using information technology approaches to evaluate the impact of public health campaigns, and (2) summarize the knowledge gained from understanding the techniques employed, variables of interest analyzed, and the metrics used to validate these results. Results of this study help identify gaps and provide a direction to appropriately position new research activities in this domain.

Mass media campaigns have long been a tool for promoting public health (6) and there are many systematic literature reviews in this domain. Some of them focus on specific public campaigns: Te et al. (7) identifies social media health campaigns against the consumption of sugary drinks; Jones and Salazar (8) describes the use of Social Network Services (SNS) in the context of primary HIV prevention; Yadav and Kobayashi (9) assesses newer evidence from quantitative studies on the effectiveness of mass media campaigns for reducing alcohol-impaired driving (AID) and alcohol-related crashes; Vega and Roland (10) describes the social marketing approaches used to increase syphilis awareness in eight US cities.

Conversely, some studies do not target a specific public, such as: Shi et al. (11) aims to differentiate SNS from more traditional health communication approaches; Jacob et al. (12) determines the costs, benefits, and overall economic value of communication campaigns; Robinson et al. (13) evaluates the effectiveness of health communication campaigns that use multiple channels, including mass media, and distribute health-related products; Randolph et al. (14) discusses the effects of three particular campaign strategies, entertainment education, law enforcement, and mass media.

Dorfman et al. (1) proposed a taxonomy of communication campaigns, which includes three axes: purpose, scope, and maturity. By purpose, the authors understand that the goal of a campaign should directly affect the individuals, or the collective policies, that shape social behavior. Scope refers to the most visible part of a communication campaign: its size and extent (In what region? In what period? Is it local, state or national?). Maturity can come over time (campaigns can be unique events or could last for years). It may become more formal, with clearer goals, well-developed materials, and deeper integration into an organization's overall activities.

This taxonomy was important to characterize a public campaign, but we missed other properties that we believe to be relevant. Such properties, presented in Table 1, in addition to Dorfman et al.'s taxonomy, helped us to identify public campaigns within the scope of our work. A catalog with the identification of the primary studies of our research is provided in Appendix 1, based on all identified properties.

**Table 1:** Properties added to the taxonomy proposed by Dorfman et al (1).

**Property**	**Reasoning**
Campaign name	The campaign's name can be able to highlight the action novelty's level. New campaigns bring similar slogans compared to previous campaigns, losing their impact.
Ads type	Detailing the advertisement type allows greater accuracy when making comparisons about the effects produced by them.
Topic area	Topic area can demonstrate that there are subjects that can have repercussions on audiences, depending on the values and social representations that they carry out in different societies, as well as knowing if a subject remains in evidence over time.
Launched/sponsor by	Understand whether a public campaign is launched by a government entity, an NGO or by the social action of a private company can produce differences in strategic and impact terms.
Amount spent	The amount spent compared to the identified impacts can be a strategic way of measuring the quality of investment in health communication actions.

## Methods

The research methodology for conducting this systematic literature review (SLR) was performed from the guidelines proposed by Kitchenham and Charters (4) and Petersen et al. (5). Although the PRISMA (Preferred Reporting Items for Systematic Reviews and Meta-Analyses) guidelines (15, 16) were not used in this study, the guidelines used follow similar stages, such as: (i) articles were identified through a database search; (ii) articles were screened according to the selection criteria concerning title and abstract; (iii) the eligibility of each full-text article was rated according to predetermined criteria (quality assessment); (iv) approved articles were included in the systematic review.

### Research Questions

The goal of this research is to analyze information technology approaches that assess the impact of public health communications campaigns as a health care promotion strategic method. Thus, we framed the research questions using the PICOC (Population, Intervention, Comparison, Outcome, and Context) criteria as suggested by Kitchenham and Charters (4).

- Population: public health campaigns.
- Intervention: the use of technology information to evaluate health campaigns.
- Comparison: characterization of the measures used to support the evaluation of health campaign.
- Outcomes: characterization of the produced output, emergent techniques, overlooked areas and open issues.
- Context: any case where technology information approaches were used to evaluate health campaign.

This leads to the following research questions (RQs):

- RQ1: When and where have the studies been published?
- RQ2: What are the characteristics (topic area, location and year) of the campaigns?
- RQ3: What variables of interest were analyzed to assess the impact of public health campaigns?
- RQ4: What techniques or tools have been used to support campaign impact analysis?

### Search Process

Scopus database was the main source of scientific papers for the current study. We selected this digital library because it contains publications from major journals and conference proceedings, and it is one of the largest curated bibliographic abstract and citation databases of research literature (17). Moreover, recent bibliographic research indicated it as the most comprehensive and user-friendly database (18, 19).

In May 2020, we conducted a preliminary search with a set of terms related to the research topics based on Population and the Intervention previous defined. The search generated many results. However, few were relevant. We repeated this process until we found a search string with a set of relevant results. To maximize recall, we did not include the comparison, outcomes, and context criteria in the search term. We considered the context criterion in Inclusion Criteria (IC), while the outcomes and the comparison criteria defined our data extraction strategy.

The set of search terms included three aspects: (i) “campaign^*^” AND (ii) (“public health” OR “communicable disease” OR “disease transmission” OR “transmitted infection” OR “disease outbreak^*^” OR “illness outbreak^*^” OR “infectious disease^*^” OR “disease surveillance” OR “disease epidemiology”) AND (iii) (“impact” OR “correlation” OR “assess” OR “effectiveness” OR “efficacy” OR “evaluation” OR “analysis”).

The automated search looked for these terms in titles, abstracts, and keywords, in the Computer Science area. To increase the quality of the studies found, they had to be published in a peer- reviewed journal. In addition, a backward snowball search (20) was performed by scanning the bibliographies of all selected papers.

### Inclusion and Exclusion Criteria

The systematic review included studies that met the following two inclusion criteria: (IC1) The study discusses the effectiveness of using information technology approaches to measure public health campaigns; (IC2) The paper evaluates a health care campaign.

Regarding the exclusion criteria (EC), studies were excluded if they presented at least one of the following criteria: (EC1) The study is not written in English; (EC2) The study is an older version of other study already considered; (EC3) The study is a short publication (<5 pages) or poster; (EC4) The study is a gray literature (For example: technical reports, white papers, and work in progress); (EC5) Paper full text is not available for download.

### Paper Selection

**Figure 1:**
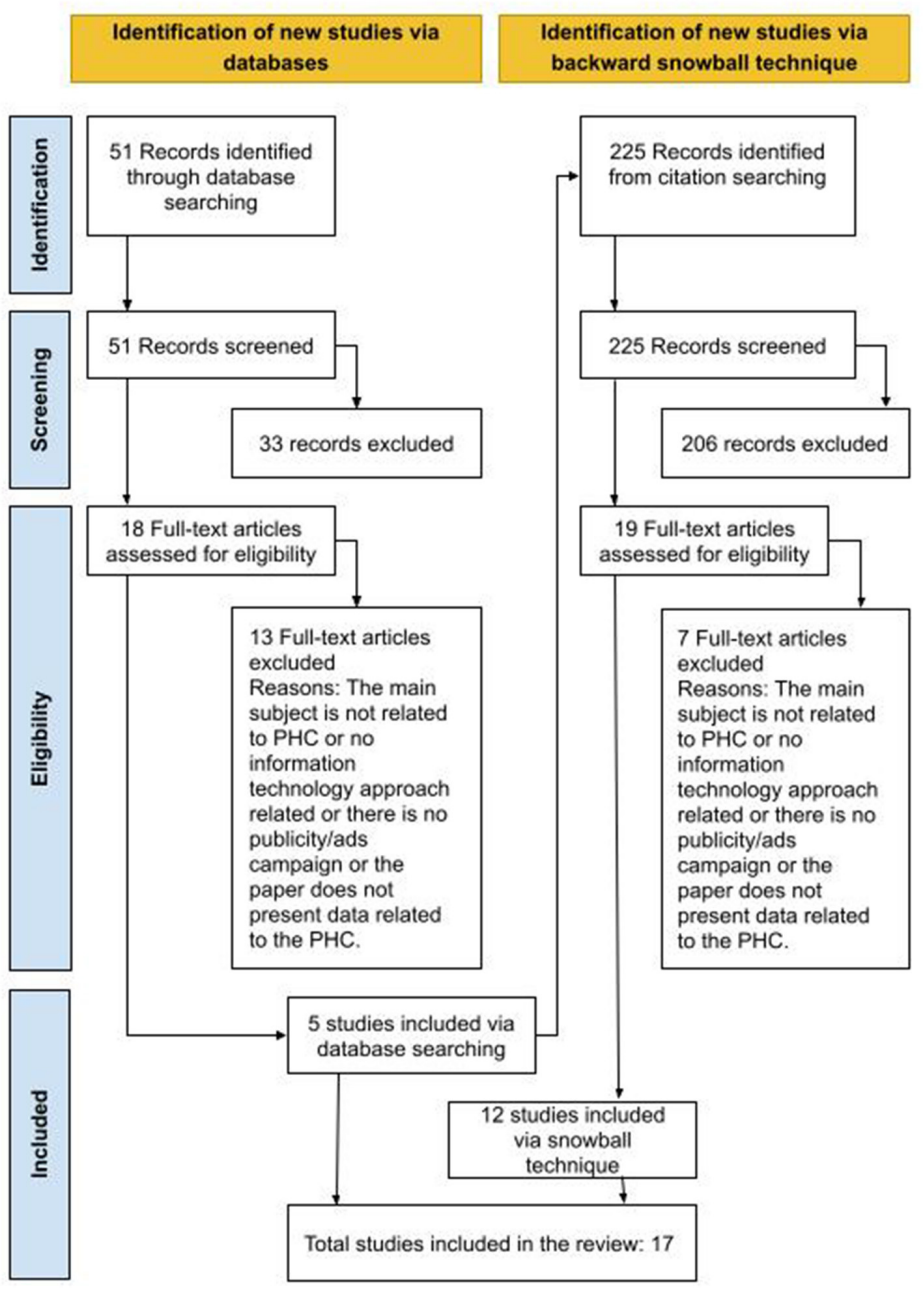
Flow diagram of the study selection process and results.

Figure 1 shows the steps and quantities of papers returned in the entire search process. The process of selection and classification of the studies was performed by the first author of this article and the second author reviewed the process to avoid research bias.

Titles and abstracts of papers resulting from the automated search were evaluated, rejecting papers that were obviously not relevant (Stage 1-identification). If eligibility was unclear from the abstract, the study was retained for further evaluation (Stage 2–screening). Subsequently, in Stage 3-eligibility, the full texts were read to confirm the criteria and submitted to Stage 4–included for data extraction.

Fifty-one Studies Were Analyzed in Stage 2 and 18 in Stage 3. However, Only five Documents Were Included for Data Extraction. A Complete Analysis of 18 Documents Was Necessary to Understand the Research Context and to Apply the Inclusion and Exclusion Criteria More accurately.

Those five documents served as basis for a subsequent analysis, through their own references, using the backward snowball technique. Thus, 225 articles were identified from citation searching (Stage 1), and screened (Stage 2) in order to verify the inclusion and exclusion criteria. In Stage 3, 19 articles were selected for complete reading and, finally, 12 articles met the determined criteria. Accordingly, 17 studies (five identified via database plus 12 identified from snowball technique) were selected for data extraction (listed in Appendix A).

### Study Quality Assessment

Quality assessment is essential in systematic reviews to determine the rigor and relevance of the primary studies and should be applied in a similar way across the different types of studies identified (21).

To assess the quality of the studies, we developed a survey on six characteristics of communication campaigns, Table 1 (3rd to 8th rows). In addition, we only consider studies classified as Evaluation Research, as defined by Wieringa et al. (22) that is: techniques, methods, tools or other solutions are implemented and evaluated in practice.

For each question, the study's quality was evaluated as “Yes,” “Partially,” or “No,” and scored with the values 1, 0.5, and 0, respectively. We noted that the overall quality of the studies was good, ranging from 0.75 to 1. Table 2 shows the quality assessment criteria found for the 17 selected studies. In Appendix A, these data are listed in detail.

**Table 2:** Quality scores.

**Questions**	**S01**	**S02**	**S03**	**S04**	**S05**	**S06**	**S07**	**S08**	**S09**	**S10**	**S11**	**S12**	**S13**	**S14**	**S15**	**S16**	**S17**
**1. Is the campaign clearly characterized?**																	
Does the campaign have a name?	1	0	1	1	1	1	0	0.5	1	1	1	1	0.5	0.5	0.5	1	1
Does the campaign have ads?	0	1	1	1	1	1	1	1	1	1	1	1	1	1	1	1	1
Does the campaign have a target audience?	1	1	1	1	1	1	1	1	1	0	1	1	1	1	1	1	1
Does the campaign have a period of time?	1	1	1	1	1	1	0	1	1	1	1	1	1	1	1	1	1
Does the campaign have a level of organization?	1	1	1	1	1	1	1	1	1	1	1	1	1	1	1	1	1
Does the campaign have a representative/sponsor?	0	1	1	1	1	1	0	0	1	1	1	1	1	1	1	1	1
Total	0.67	0.83	1.00	1.00	1.00	1.00	0.50	0.80	1.00	0.83	1.00	1.00	1.00	1.00	1.00	1.00	1.00
**2. Is the scheme classification clearly defined as evaluation research?**																	
Are techniques, methods, tools or other solutions evaluated in practice?	1	1	1	1	1	1	1	1	1	1	1	1	1	1	1	1	1
Are the outcomes investigated?	1	1	1	1	1	1	1	1	1	1	1	1	1	1	1	1	1
Total	1	1	1	1	1	1	1	1	1	1	1	1	1	1	1	1	1
Overall quality	0.83	0.92	1.00	1.00	1.00	1.00	0.75	0.90	1.00	0.92	1.00	1.00	1.00	1.00	1.00	1.00	1.00

### Data Extraction

To extract data from the identified primary studies, we developed a template (Table 3) to register the main information in a spreadsheet. Each data item addresses a Research Question (RQ), Quality Assessment (QA) or an overview of the study.

**Table 3:** Data extraction form.

	**Data item**	**Description**	**RQ**
Paper	Paper ID	Integer	Overview
	Title	Name of the paper	Overview
	Author	Set of names of the authors	Overview
	Country	First author country	RQ1
	Venue	Name of publication venue	RQ1
	Year	Year of publication	RQ1
Campaign	Campaign name	Name/Title of the campaign	Overview/QA
	Ads type	Ads type used in the campaign	Overview/QA
	Topic area	Topic area addressed in the campaign	RQ2
	Target audience	General Public; Target Public; Policy-makers	Overview/QA
	Country campaign	Country where the campaign was carried out	RQ2
	Period of time	Campaign duration period	RQ2
	Level of organization	Local, state or national	Overview/QA
	Launched/sponsor by	Representative who launched or sponsored the campaign	Overview/QA
	Amount spent	Total amount spent in Campaign	Overview
Evaluation	Data sources	Data sources explored by the study	RQ3/QA
	Data source category	Category that best characterizes the data source	RQ3/QA
	Variables of interest	Variables of interest explored by the study	RQ3/QA
	Dimension	Area related to the variable of interest identified	RQ3
	Tools or technologies	Techniques, methods, tools or other solutions implemented	RQ4/QA
	Results	Main results reported	Overview

### Threats to Validity

The ability to choose digital libraries that will accurately represent a study can influence the validity of an SLR. Scopus has one of the largest abstracts and citation databases for research literature. Researchers consider it a high-quality, versatile, and respected research database (19).

This review created more restrictive filters for the area (Science Computer) and source type (Journals), to avoid an exhaustive review. Accordingly, some studies–although relevant–may not be included. We mitigated this limitation by scanning the reference list through a backward snowballing technique (20) of identified papers. This technique added 225 papers for the analysis of titles and abstracts, of which 19 were included in the final selection.

Regarding data extraction, the process of selection and classification of the studies was done by one researcher and all data extracted was checked by the other, and subsequently rechecked by the first researcher. At the end, we discussed the results and when doubts remained, a third researcher was consulted, to make the final decision.

## Results

In total, 276 papers were found by the automatic and snowball searches. However, after applying the inclusion/exclusion criteria, 17 papers remained in the set of relevant papers. We believe that these 17 papers present the necessary criteria to answer the research questions.

**RQ1: When and where have the studies been published?** Historically, the first publication was in 2003. Then, in 2008. After 3 years with no results found, publications returned in 2011, where there were annual publications until 2019. The countries carrying out this research were: Australia (*n* = 6), United States (*n* = 6), United Kingdom (*n* = 3), China (*n* = 1), and Norway (*n* = 1). Most studies were published in health and communication journals as shown in Table 4.

**Table 4:** Sources identified in primary studies, ordered by year.

**Year**	**Author country**	**Venue name**	**Cite**	**Study**
2003	United States	Health communication	(23)	S07
2008	Australia	American journal of public health	(24)	S06
2011	Australia	Sexual health	(25)	S15
2012	United States	American journal of preventive medicine	(26)	S08
2013	Australia	Sexual health	(27)	S13
2014	United Kingdom	Journal of medical internet research	(28)	S11
2014	United States	Journal of communication	(29)	S09
2015	United Kingdom	Data mining and knowledge discovery	(30)	S01
2015	United States	AIDS and Behavior	(31)	S12
2016	United States	JMIR public health surveill	(32)	S14
2016	Australia	Journal of health communication	(33)	S16
2017	United States	Tobacco control	(34)	S10
2017	Norway	International journal of e-health and medical communications	(35)	S02
2017	China	IEEE transactions on nanobioscience	(36)	S03
2018	Australia	Australian and New Zealand journal of public health	(37)	S17
2018	Australia	Social media and society	(38)	S05
2019	United Kingdom	Digital health	(39)	S04

**RQ2: Which topic areas were addressed to the campaign, location, and year?** In the 17 selected studies, the topic areas addressed were Anti-Smoking [*n* = 5, (S03, S06, S07, S09, S10)], Children Vaccination [*n* = 1, (S01)], Newborn screening and biobanking programs [*n* = 1, (S14)], Overweight and Obesity [*n* = 3, (S05, S16, S17)], and Sexual Health Care [*n* = 7, (S02, S04, S08, S11, S12, S13, S15)].

**Figure 2:**
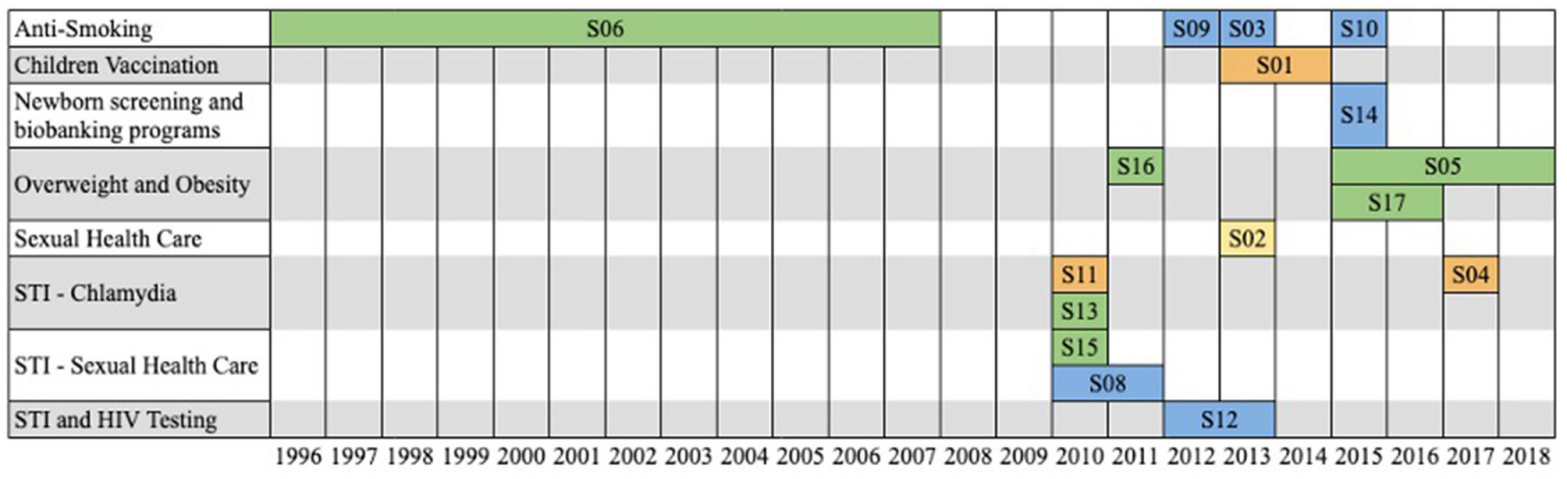
Distribution of topic areas addressed to campaign by range of period.

Figure 2 shows the distribution of thematic areas targeted to the campaign in the period from 1995 to 2018. Each rectangle covers a period on a campaign topic by country. Each country is designated by a color. Australia (green) covered long-term campaigns in the Anti-Smoking, Overweight and Obesity, and Sexual Health Care areas. The United States (blue) had campaigns in the areas of Anti-smoking, Newborn screening and biobanking programs, and Sexual Health Care. England (orange) in Sexual Health Care (Chlamydia) and Children Vaccination. Norway (yellow) had a short-term campaign in the Sexual Health Care area.

When the dates were not clearly specified in the article, an internet search was carried out to find approximate values. For example: In S01, influenza season (2013/2014) meant the period from 02/09/2013 to 13/04/2014 (It was dominated by the circulation of influenza A(H1N1) pdm09 virus, in Europe). When the articles specified only the month and year of the campaign, we considered the first (starting) and last (end) day of the month. One study (S07) did not report a period and due to this it was not shown in the Figure 2.

**RQ3: What variables of interest were analyzed to assess the impact of public health campaigns?** The 17 studies evaluated several types of variables, such as: amount of access to the campaign website, awareness and knowledge of the subject through the questionnaires, interest of the population through the online search engine, engagement through the SNS, and advertising exposure data of television commercials.

Most studies sought to cross-check the data obtained with socioeconomic and census data. The data source used to obtain the variables can be categorized as follows: questionnaire (*n* = 10), social network service (*n* = 10), Sexually Transmitted Infection (STI) testing (*n* = 2), television commercials (*n* = 2), campaign website (*n* = 1), pharmaceutical products (*n* = 1), smoke-free restaurant laws (*n* = 1), sociodemographic data (*n* = 1), tobacco prices (*n* = 1). Table 5 shows data sources, variables of interest, dimensions, and methods used by each study.

**Table 5:** Data sources and variables of interest analyzed per study.

**Paper**	**Data source**	**Variables of interest**	**Dimension**	**Method used**
**S01**	Search engine	Bing search queries geo-located in the target vaccinated locations.	Communication	The authors presented a statistical framework for estimating the prevalence of an intervention campaign in the population from Internet data.
	Social network service	Number of Twitter postings.	Communication	
**S02**	Social network service	Number of visitors/visits, time spent.	Communication	The authors analyzed the impact of a Facebook fan page, a Facebook advertisement campaign, and posters through Facebook statistics dashboard and Google Analytics.
**S03**	Television commercials	Tweets related to the televised ads.	Communication	The authors presented a statistical framework (Advertising Social Influence Estimation-ASIE) which predict the probability of users posting tweets influenced by both TV broadcasting and friends in the online social network.
	Social network service		Communication	
**S04**	Questionnaire	Questions about attitudes toward sexual health promotion on social media, preferences for the content of promotional campaigns, and potential barriers to engagement with social media health promotion.	Epidemiology	The authors analyzed qualitative face-to-face interviews, and the engagement with chlamydia testing page through Facebook statistics dashboard and Google Analytics, as well as descriptive statistics to assess amount of ? chlamydia tests requested during the intervention period.
	Social network service	Number of visitors/visits and online actions.	Communication	
	STI testing	Number of chlamydia tests requested during the campaign period.	Epidemiology	
**S05**	Questionnaire	Questions about the experience and satisfaction with a health campaign in Facebook. Participants' self-reported postcode, and height and weight.	Communication	The authors conducted statistical tests to compare Facebook users' groups and explored its profile to examine the characteristics of fans of the page, as well as analyzed an online survey to investigate how users were interacting with the campaign page and others health pages on Facebook.
**S06**	Questionnaire	Smoking prevalence was estimated from survey.	Epidemiology	The group used time-series autoregressive integrated moving average analysis in a statistical software (SAS) to estimate the effect of antitobacco advertising and tobacco policies on monthly smoking prevalence.
	Television commercials	Occurrences of all tobacco-related advertisements appearing on television.	Communication	
	Tobacco prices	Cigarette costliness was measured with the ratio of the average recommended retail price per cigarette pack to the average weekly earnings.	Public policy	
	Pharmaceutical products	Population use of pharmaceutical smoking cessation products.	Epidemiology	
	Smoke-free restaurant laws	Population exposure to smoke-free laws was expressed as the percentage of the total sample that was subject to such laws.	Public policy	
**S07**	Questionnaire	Questions about the participants perception related to campaign messages and their behavioral intent post- campaign.	Communication	The authors conducted statistical tests to analyze three primary variables of interest (source evaluation, message evaluation, and behavioral intention), based on the theory of psychological reactance.
**S08**	Questionnaire	Questions about sexual health care behavior.	Epidemiology	The authors analyzed data related to the engagement with the Facebook intervention page through Google Analytics and an online survey, using a statistical software (SAS).
	Social network service	Engagement with the Facebook page.	Communication	
**S09**	Social network service	Message acceptance, rejection, and disregard from each tweet identified as Tips-relevant.	Communication	The authors present an analysis of Twitter messages about a health campaign using an analytic framework.
**S10**	Social network service	Messages were labeled as anti, pro, or neutral campaign.	Communication	The authors analyzed the content of Twitter messages about a health campaign through statistical analysis.
**S11**	Social network service	Volume of interaction. The total number of fans, wall posts, and comments over time, fan demographics. Website access numbers and viewing patterns.	Communication	The authors examined quantitative and qualitative data on a Facebook page about a health campaign. Google analytics was used to describe the number of people using the page and viewing patterns.
**S12**	Social network service	Data related to numbers of “likes,” visits, and number of “followers.”	Communication	The group used standard descriptive statistics to assess a health campaign by: tracking website/social media use, online survey, and comparing rates of STI testing.
	Campaign website	Data related to the engagement on campaign website.	Communication	
	Questionnaire	Information on age, how they heard about the campaign, assessed knowledge of STIs, and if the campaign influenced intention to get tested.	Communication and Epidemiology	
	STI testing	Checking STI Testing Pre- and Post-Campaign.	Epidemiology	
**S13**	Questionnaire	Questions about demographic variables, height and weight, how they found out about the study, sexual history, experience and knowledge of STIs.	Epidemiology	The group assessed the feasibility of using SNSs to recruit young women to complete a health-related survey.? Data were analyzed using a statistical software (STATA).
**S14**	Social network service	Engagement with the Facebook: Page likes, views, posts and photo album engagement (likes, connections, shares, conversation, and comments).	Communication	The authors presented a framework for examining a spectrum of Facebook engagement outcomes from observation to conversation. Data were provided by Facebook dashboard.
**S15**	Questionnaire	Number of clients that contacted the service after ads exposure and ads type used.	Communication	The authors presented a simple descriptive study evaluating the effectiveness of different advertising methods.
**S16**	Questionnaire	Questions about demographic variables, risk factors, if the respondent was aware of the “Swap It” campaign, attitudes and behaviors regarding diet and exercise, and behavioral intentions and actions.	Communication and epidemiology	The authors evaluated a health campaign via cross-sectional serial telephone surveys. Data were analyzed using a statistical software (STATA).
**S17**	Questionnaire	Questions about campaign awareness, knowledge, attitudes, and intentions. Current behavior and recent behavior change. Demographic variables, body mass index category and risk index score.	Communication and Epidemiology	The authors presented a cohort design study and sed generalized linear mixed models in a statistical software (SAS) to examine campaign awareness, knowledge, attitudes, intentions, and behaviors over time

Despite the variety of variables analyzed, we can observe a predominance related to i) assessing user engagement with the campaign subject through SNS, such as Facebook and Twitter (S01, S02, S03, S04, S08, S09, S10, S11, S12, S14); and ii) assessing public knowledge, attitudes and behavior through questionnaires (S04, S05, S06, S07, S08, S12, S13, S15, S16, S17), before and/or after campaign dissemination.

SNS were used to recruit participants to answer the questionnaires (S05, S08, S13), assessing the reach and knowledge of the impacted people. The authors argued that this approach produced good results due to accurate user geolocation and demographic data (e.g., gender, age, education, employment status, language spoken).

In addition, some studies (S02, S04, S05, S08, S12, S14) highlighted that Facebook advertisements significantly helped to increase both the number of people visiting a campaign webpage, and the number of users who could potentially improve their knowledge and health care behavior, because of the campaign.

Televised ads were assessed in two anti-tobacco campaigns using broadcasting time and its Nielsen rating (https://www.nielsen.com/ca/en/about-us/). S03 used TV ratings correlated with social media (Twitter) while S06 correlated TV ratings with questionnaire, tobacco prices, population use of pharmaceutical smoking cessation products, and smoke-free restaurant laws. In both cases, the results indicated success in the approaches used to assess the campaign impact.

Regarding STI Testing, S04 and S12 indicate that their campaign achieved its goal, i.e., to change sex care behavior and reach a large number of people. In fact, in the post-campaign period, the demand for test orders increased.

Grouping the variables of interest in dimensions (areas of context) provided better understanding about which dimension was most explored. Our findings show three dimensions: communication (*n* = 20), epidemiology (*n* = 10), and public policy (*n* = 2).

Communication dimension was assigned when the variable of interest sought to assess engagement on the Internet/social media or surveys asked the user knowledge about the health campaign. Epidemiology dimension was assigned when the variable of interest sought to assess patterns of health and disease conditions in a defined population. Public policy dimension was detected when the variable of interest sought to assess control policies, such as changes in taxes on products, and new laws.

**RQ4: What techniques or tools have been used to support campaign impact analysis?** Considering that social networking services (Facebook, Twitter, and Instagram) were widely explored in the studies, the main tools were the dashboards of the respective sites (explored by S02, S04, S08, S11, S12, S14). SNS dashboards often provide enough data to verify the engagement of privately hosted websites.

Nevertheless, analyzing user-generated content on a social network, such as Twitter, and inferring correlations with campaign data requires a detailed evaluation of the content. Accordingly, the studies S03, S09, and S10 obtained data from Twitter through a licensed data provider (http://www.gnip.com). This vendor provides real-time access to 100% of all tweets and meta-data. These analyses and correlations were supported by general linear models used to assess the relationship between demographics data and campaign awareness. Furthermore, Google analytics was used to identify the number of unique users, total visits, page views, unique views, and average visit duration for each page of the campaign website.

Regarding software for statistical analysis and using data science to explore, visualize, model and make inferences in the data, studies S12, S13 and S16 used Stata (https://www.stata.com/) while studies S06, S08, and S17 used Statistical Analysis System–SAS (https://www.sas.com/enca/software/stat.html). NVivo (https://www.qsrinternational.com/nvivo/home), a qualitative data analysis software, was used by S05 for textual analysis of the questionnaires and by S11 to capture the campaign page content, using a simple counting method to describe user and moderator content, discussion thread patterns, and moderator intervention. S12 used a web-based survey administration and data management application called REDCap (https://www.project-redcap.org/).

In S05, all statistics analyzes were conducted with SPSS Statistics 22 (https://www.ibm.com/support/pages/spss-statistics-220-available-download), a software platform that offers advanced statistical analysis, a vast library of machine learning algorithms, text analysis, open-source extensibility, integration with big data and seamless deployment into applications.

S01 proposed a statistical framework for transforming user-generated content published on web platforms to an assessment of the impact of a health-oriented intervention and S07 used a statistical technique named ANOVA (analysis of variance) to test all variables defined in the study.

## Discussion

This paper assessed how researchers evaluate public health campaigns. Based on the studies found and the answers to the research questions, we observed that the studies sought to assess campaign impact through quantitative and qualitative analysis, using mainly SNS, especially Facebook. The popularity of social platforms such as Facebook–in the United States and worldwide–serves to justify this approach (40, 41). Facebook ads can reach and engage large, well-specified populations at relatively low cost (42, 43).

In addition, SNS are a rich source of data that can provide responses to PHC impact assessments. This may be because data is available to users through the dashboards, where it is possible to observe quantitative values. Detailed data can be easily obtained from some vendors, by paying fees, which facilitates data collection, allowing such methods and tools to do the “heavy lifting” (sometimes paid, such as Stata, SAS, NVivo or SPSS). A potential threat related to this approach is to identify real engagement of users. Some studies reported this issue (S4, S5, S8, S11, S14), which raised the following question: What is the real motivation to engage on campaign interventions pages on social networks? What about the “spiral of the silence” (44) which suggests that interventions can change the attitudes and behaviors of real interested users behind the screen, even without their interaction?

It is intriguing to consider the paradox of trying to reach a target audience through the SNS to raise awareness about an infectious disease, since some parts of the population cannot be reached through technologies and the Internet. In Brazil, for example, internet use reached 152 million people, representing 81% of the population. This percentage drops to 67% when we looked at the lower social classes (45). However, at the same time, it is likely that no communication channel will be able to reach 100% of the target audience by itself. The technological bias present in many campaigns is clear when only digital channels are activated, mainly excluding lower social classes, homeless people and people deprived of liberty, who in turn are part of the target audience of this type of campaign.

In addition, questionnaires targeting impacted users was another effective form of evaluating campaign impact. This approach is useful for measuring target audience awareness, attitudes and behavior, and has been used in eight out of 17 primary studies.

In contrast to most of the studies analyzed, article S15 reported that advertisements through paper-based methods, text messaging and social networking sites were not effective. In this study, only 28 rural youth contacted the health care service (campaign's goal) over the 11-month period. Twenty young people were reached by nurses (15 from school nurses, five from community health nurses), six through the campaign webpage, one through Facebook and one through the student diary. No clients were recruited through other advertising methods. Arguably, the limited publicity could be because the target audience were young people living in rural areas in Victoria, Australia. Therefore, although SNS have a great potential to provide data to analyze the impact of campaigns, attention must be paid to the public and the conditions of access and web-behavior of that public.

Despite the widespread use of social networking sites, only one study (S01) sought a second source of data based on user-generated Internet content (Microsoft's Bing search engine). The study aimed to introduce a complementary framework for evaluating the impact of a targeted intervention, such as a vaccination campaign against an infectious disease, through statistical analysis of user-generated content submitted on web platforms (Twitter and Bing search engine). The results from Twitter data demonstrated less sensitivity across similar controls relative to Bing data, suggesting a greater reliability.

In general, we observed a gap in assessing the impact of public health campaigns, regarding the use of online data (i.e., online news) and others user-generated Internet content. Google Search and Yahoo should be used in addition to Bing search engine to check the increase of news related to the campaign. In addition, a variety of questions should be asked, such as what can the analysis of this content bring? Are campaigns helping to grow spontaneous news on the related topic? When evaluating a population over time, Google Trends is arguably an excellent tool to demonstrate how the population has sought to know about a certain topic (46–48). However, it was not explored in the context of these works.

Moreover, databases of scientific papers such as Springer Nature, Wiley Blackwell, Taylor and Francis, IEEE, American Physical Science and Elsevier and its indexers such as Scopus, Web of Science (WoS) and Google Scholar could be used to demonstrate the interest of the academy in developing research on the campaign theme in a comparison of time (before, during and after the campaign). These variables of interest can show a new dimension, Education.

Health campaigns are common worldwide. Nevertheless, few studies have reported concrete campaign data. We would like to encourage future research to better specify analyzed data in their studies showing properties raised by Dorfman et al. (1) and complemented for us, to enable a comparison on health campaigns analysis, as present in Appendix A, stimulating the Campaign dimension.

Notably, by using variables of interest that were grouped into three dimensions (as discussed in this review), we analyzed the reach of the “Syphilis No!” campaign (49), assessing data related to the campaign, online news, search engine activity, online courses, serological tests, medication distribution and case notification rates. Results of this analysis show positive changes over time in communication, education, and epidemiological surveillance dimensions, especially after the campaign propagation.

## Conclusions

The paper provided an overview of studies that evaluate the impact of public health campaigns. The analysis focused on identifying variables of interest, techniques and tools used. We discussed the results, presented new questions, and discovered unexplored variables of interest.

Public health campaigns play a strategic role promoting awareness, increasing knowledge, and encouraging the target population to adopt desirable attitudes and behaviors. As observed, its impact must be measured in several dimensions such as: i) communication (engagement in social networks, questionnaires assessing the user's knowledge about the campaign), ii) epidemiology (disease screening test, cases' notification, questionnaires assessing the user's knowledge about the disease/health issues), and iii) policy enforcement (law strategies on health promotion).

This multidimensional analysis provided a complete evaluation of public health campaigns, to understand its scope, find correlations between different variables of interest and expand possibilities for analysis.

Accordingly, we hope this work will motivate future researchers to explore other variables of interest and dimensions that are possibly overlooked in assessing the impact of public health campaigns based on multidimensional aspects.

## Data Availability Statement

The original contributions presented in the study are included in the article/Supplementary Material, further inquiries can be directed to the corresponding author.

## Author Contributions

RP, LS, and VK: conceptualization. RP, LS, RV, and VK: methodology. RP, LS, RV, VK, CG, CO, and JL: validation and writing–review and editing. RP and LS: formal analysis. RP: investigation, data curation, and writing–original draft preparation. LS, RV, and VK: supervision. RV: project administration and funding acquisition. All authors contributed to the article and approved the submitted version.

## Funding

Brazilian Ministry of Health–Number's project: 54/2017.

## Conflict of Interest

The authors declare that the research was conducted in the absence of any commercial or financial relationships that could be construed as a potential conflict of interest.

## Publisher's Note

All claims expressed in this article are solely those of the authors and do not necessarily represent those of their affiliated organizations, or those of the publisher, the editors and the reviewers. Any product that may be evaluated in this article, or claim that may be made by its manufacturer, is not guaranteed or endorsed by the publisher.
