# Supplementary material for: Systematic Review on Information Technology Approaches to Evaluate the Impact of Public Health Campaigns: Real Cases and Possible Directions
Source: Front Public Health. 2022 Jan 11;9:715403. doi: 10.3389/fpubh.2021.715403 (PMC8787277; doi:10.3389/fpubh.2021.715403)
Supplement: Supplementary file 1 [file Data_Sheet_1.docx]

Supplementary Material

**Appendix A**

In this appendix, we present the identification of the primary studies of our research: title and campaign data.

S01 - Assessing the impact of a health intervention via user-generated Internet content (Lampos et al., 2015)

- Campaign Name: Live Attenuated Influenza Vaccine
- Ads Type: Not reported
- Topic Area: Children Vaccination
- Country Campaign Location: England
- Target Audience: Target Public
- Period: 2013/2014 influenza season
- Level of Organization: National
- Launched by: Not reported
- Amount spent: Not reported
- Data Sources: Microsoft’s Bing; Twitter data

S02 - Impact of Facebook ads for sexual health promotion via an educational web app: A case study (Gabarron et al., 2017)

- Campaign Name: Not reported
- Ads Type: Facebook Ads Campaign; Posters
- Topic Area: Sexual Health Care
- Country Campaign Location: Norway
- Target Audience: Target Public
- Period: 4th April to 3rd May 2013
- Level of Organization: Local
- Launched by: University Hospital of North Norway
- Amount spent: 386 USD
- Data Sources: Facebook Data

S03 - Inferring Social Influence of Anti-Tobacco Mass Media Campaign (Zhan et al., 2017)

- Campaign Name: a) Tips from Former Smokers 2013; b) “Legacy Truth”
- Ads Type: Television Ads
- Topic Area: Anti-Smoking
- Country Campaign Location: United States
- Target Audience: Target Public
- Period: a) Mar, 1st to Jun, 23 2013; b) Aug, 11 to Oct, 28 2013
- Level of Organization: National
- Launched by: a) Centers for Disease Control; b) American Legacy Foundation
- Amount spent: Not reported
- Data Sources: TV Ads Data; Twitter Data

S04 - Targeted advertisement of chlamydia screening on social media: A mixed-methods analysis (Nadarzynski et al., 2019)

- Campaign Name: Lets Talk About It (STI)
- Ads Type: a) Posters; b) Facebook Ads Campaign
- Topic Area: Chlamydia - STI
- Country Campaign Location: England
- Target Audience: Target Public
- Period: a) Jan and Mar 2017; b) Aug, 4 to Oct, 11 2017
- Level of Organization: Local
- Launched by: a) University of Southampton b) Solent NHS Trust Clinical Governance
- Amount spent: 1,300 USD
- Data Sources: Semi-structured interviews; Facebook

S05 - User Perceptions of the Make Healthy Normal campaign Facebook Page: A Mixed Methods Study (Kite, McGill, et al., 2018)

- Campaign Name: Make Healthy Normal (MHN)
- Ads Type: TVs commercials supported by community events, press, out-of-home, on-line ads, public relations, a website and social media.
- Topic Area: Overweight and Obesity
- Country Campaign Location: Australia
- Target Audience: a) General Public; b)Target Public
- Period: a) 2015-2016; b) 2016-2018
- Level of Organization: State
- Launched by: Australian Government
- Amount spent: Not reported
- Data Sources: Online survey with participants recruited via Facebook Ads and posts on the MHN page; Facebook Data

S06 - Impact of tobacco control policies and mass media campaigns on monthly adult smoking prevalence (Wakefield et al., 2008)

- Campaign Name: Australia’s National Tobacco Campaign (between 1996 to 2001) and a state-level tobacco control programs (since 2002)
- Ads Type: Television commercials
- Topic Area: Anti-Smoking
- Country Campaign Location: Australia
- Target Audience: Target Public
- Period: From June 1995 to December 2006
- Level of Organization: National; State
- Launched by: Australian Government
- Amount spent: Not reported
- Data Sources: Survey, TV Ads Data, cigarette costliness, monthly sales of Nicotine Replacement Therapy and bupropion, and smoke-free laws.

S07 - Adolescent reactance and anti-smoking campaigns: A theoretical approach (Grandpre et al., 2003)

- Campaign Name: Not reported
- Ads Type: Messages utilizing interactive multimedia computer programs to merge photographic, music, video, audio voice-over, and text
- Topic Area: Anti-Smoking
- Country Campaign Location: United States
- Target Audience: Target Public
- Period: Not reported
- Level of Organization: Local
- Launched by: Not reported
- Amount spent: Not reported
- Data Sources: Online survey with students

S08 - Social media-delivered sexual health intervention: A cluster randomized controlled trial (Bull et al. 2012)

- Campaign Name: Just/Us (intervention page)
- Ads Type: Facebook Ads campaign
- Topic Area: Sexual Health Care - STI
- Country Campaign Location: United States
- Target Audience: Target Public
- Period: Oct 2010 and May 2011
- Level of Organization: Local
- Launched by: Not reported
- Amount spent: Not reported
- Data Sources: Survey; Facebook Data

S09 - Are You Scared Yet? Evaluating Fear Appeal Messages in Tweets About the Tips Campaign (Emery et al., 2014)

- Campaign Name: Tips From Former Smokers
- Ads Type: Billboards, TV Ads, radio, print & websites
- Topic Area: Anti-Smoking
- Country Campaign Location: United States
- Target Audience: Target Public
- Period: March 15, 2012 to June 9, 2012
- Level of Organization: National
- Launched by: Centers for Disease Control
- Amount spent: US$ 54 million
- Data Sources: Twitter Data

S10 - Campaigns and counter campaigns: Reactions on Twitter to e-cigarette education (Allem et al., 2017)

- Campaign Name: Still Blowing Smoke
- Ads Type: TV Ads, billboards, and a website
- Topic Area: Anti-Smoking
- Country Campaign Location: United States
- Target Audience: N/A
- Period: 23 March of 2015 to June 2015
- Level of Organization: State
- Launched by: California Department of Public Health
- Amount spent: Not reported
- Data Sources: Twitter Data

S11 - Would you tell everyone this? Facebook conversations as health promotion interventions (Syred et al., 2014)

- Campaign Name: Say Yes to the Test
- Ads Type: Facebook advertising campaign
- Topic Area: Chlamydia - STI
- Country Campaign Location: England
- Target Audience: Target Public
- Period: January and June 2010
- Level of Organization: National
- Launched by: English Department of Health and the Health Protection Agency (now Public Health England)
- Amount spent: Not reported
- Data Sources: Facebook Data

S12 - IknowUshould2: Feasibility of a Youth-Driven Social Media Campaign to Promote STI and HIV Testing Among Adolescents in Philadelphia (Dowshen et al., 2015)

- Campaign Name: IknowUshould2
- Ads Type: Print Ads, t-shirts, radio, website, Facebook, Twitter, Instagram, YouTube, campaign events, and community outreach & partnership.
- Topic Area: STI and HIV Testing
- Country Campaign Location: United States
- Target Audience: Target Public
- Period: September 2012 - August 2013
- Level of Organization: Local
- Launched by: Children’s Hospital of Philadelphia
- Amount spent: Not reported
- Data Sources: Facebook, Twitter, Instagram, Website campaign page

S13 - Attitudes to chlamydia screening elicited using the social networking site Facebook for subject recruitment (Ahmed et al., 2013)

- Campaign Name: (component of) Young Female Health Initiative
- Ads Type: Facebook Ads campaign
- Topic Area: Chlamydia - STI
- Country Campaign Location: Australia
- Target Audience: Target Public
- Period: May and September 2010
- Level of Organization: State
- Launched by: Young Female Health Initiative
- Data Sources: Facebook Data, Australian Bureau of Statistics 2006 Census data;
- Amount spent: Not reported
- Socioeconomic Indexes for Areas (SEIFA).

S14 - Facebook advertising across an engagement spectrum: A case example for public health communication (Platt et al., 2016)

- Campaign Name: Michigan BioTrust for Health (BioTrust)
- Ads Type: Facebook advertising campaign
- Topic Area: The newborn screening and large population biobanking programs
- Country Campaign Location: United States
- Target Audience: Target Public
- Period: Spring of 2015, 11-week
- Level of Organization: State
- Launched by: University of Michigan’s Life Sciences and Society Program
- Amount spent: US$ 15,000
- Data Sources: Facebook Data

S15 - Advertising sexual health services that provide sexually transmissible infection screening for rural young people - What works and what doesn't (Gamage et al., 2011)

- Campaign Name: TESTme program
- Ads Type: Website, Facebook page, posters, flyers, business cards, wristbands and regional awareness programs for health care. Newspapers, youth magazines and newsletters, ads in student diaries at universities and short message ads to mobile phones.
- Topic Area: Sexual Health Care - STI
- Country Campaign Location: Australia
- Target Audience: Target Public
- Period: August 2009 to June 2010
- Level of Organization: State
- Launched by: Melbourne Sexual Health Centre (MSHC)
- Amount spent: $ 20,850 (currency not specified)
- Data Sources: Survey. Clients that contacted the service after ads exposure

S16 - Impact of the Swap It, Don’t Stop It Australian National Mass Media Campaign on Promoting Small Changes to Lifestyle Behaviors (O’Hara et al., 2016)

- Campaign Name: Swap It, Don’t Stop It
- Ads Type: TV ads, radio, magazines, online settings, and out-of-home
- Topic Area: Overweight and Obesity
- Country Campaign Location: Australia
- Target Audience: Target Public
- Period: Mar to Jun 2011 and Sep to Dec 2011
- Level of Organization: National
- Launched by: Australian Government
- Amount spent: Not reported
- Data Sources: Telephone surveys.

S17 - Impact of the make healthy normal mass media campaign (Phase 1) on knowledge, attitudes and behaviours: A cohort study (Kite, Gale, et al., 2018)

- Campaign Name: Make Healthy Normal (MHN)
- Ads Type: Television commercials supported by community events, press, out-of-home (e.g. billboards), online advertising, public relations, a website and social media.
- Topic Area: Overweight and Obesity
- Country Campaign Location: Australia
- Target Audience: Target Public
- Period: Phase 1) June 2015 to June 2016;
- Level of Organization: State
- Launched by: Australian Government
- Amount spent: Approximately $AU 3.5 million, with $AU 2.6 million allocated to cover the media costs, with the remainder for evaluation and research, creative design and production.
- Data Sources: Online surveys with participants recruited via a research panel
